# Evaluation of obturation techniques in primary teeth among Indians

**DOI:** 10.6026/973206300191324

**Published:** 2023-12-31

**Authors:** Karuna Sharma, Rahul Maria, Smita Durga Dutta, Mansi Semwal, Kodali Srija, Madhura Pawar, Namrata Dagli, Dhaval Niranjan Mehta

**Affiliations:** 1 Department of Pediatric and Preventive Dentistry, Rama Dental College, Hospital and Research Centre, Kanpur, U.P., India; 2 Department of Conservative Dentistry and Endodontics, Jaipur Dental College, Jaipur, Rajasthan, India; 3 Department of Conservative Dental Sciences and Endodontics, Qassim University, Saudi Arabia; 4 Department of Pediatric and Preventive Dentistry, Babu Banarasi Das College of Dental Sciences, BBDU, Lucknow, India; 5 Department of Pediatric and Preventive Dentistry, Supraja Dental Clinic, Moti Ngar, Hyderabad, Telangana, India; 6 Department of Pediatric and Preventive Dentistry, Dr D Y Patil Dental College and Hospital, Dr D Y Patil Vidyapeeth, Pimpri, Pune, India; 7 Karnavati Scientific Research Centre, Karnavati School of Dentistry, Karnavati University, Gandhinagar, India; 8 Department of Oral Medicine and Radiology, Narsinbhai Patel Dental college and Hospital, Sankalchand Patel University, Visnagar, Gujarat, India

**Keywords:** Endodontic pressure syringe, reamers, incremental filling technique, obturation, primary teeth

## Abstract

Evaluation of four distinct obturating methods namely endodontic pressure syringe (n=40), reamers (n=40), Lentulo spirals driven through slow-speed handpiece (n=40) and incremental filling technique (n=4) using zinc oxide eugenol (ZOE) paste as obturating material in deciduous teeth is of interest to dentist. Hence, we are interested determining the effective obturation methodology using CBCT. Handpiece driven lentuspirals helped in optimum obturation in high percentage of root canals. Low percentage of optimally filled root canals was observed in reamers technique. Moreover, under filled root canals was low in lentuspirals technique of obturation. Thus, overfilled root canals were high in endodontic pressure syringe and reamers obturation technique.

## Background:

Pain relief was the sole justification for pulpal treatment in the past [1,2]. Endodontics plays a larger role in ensuring the survival of optimally functioning dental pulp as a result of scientific advancements in the field of dental care [3,4]. Since deciduous teeth are among the most effective space maintainers, it is crucial to preserve the structural integrity of the arch's design by keeping the primary dentition unaltered until the emergence of the permanent successors [5,6]. Aseptic root canal preparation and root canal system sealing are essential for successful endodontic therapy [7, 8]. Owing to their uneven and complicated structure, primary teeth's root canals present a challenge for achieving the perfect biomechanical endodontic intervention. Studies are being done to determine the best obturating materials and methods for the unique characteristics of root canals in primary teeth [9, 10]. Several approaches have been used to obturate decidous teeth: the manual incremental technique, different injection or syringe approaches, the lateral condensation procedure utilising pluggers or spreaders and the manual and rotating Lentulo spiral method [11,12]. Numerous writers have investigated the efficacy of different obturation methods, in vitro as well as in vivo, such as digital radiography, radiographs, radioisotopes and dyes and fluid filtration [13,14 15]. However, no method fulfils all the requirements of the perfect approach, nor does it assess obturation in all three directions [16-17, 18].

More recently, studies conducted in vitro have employed CBCT to rapidly and precisely recognize voids and analyse the shape of root canal of the primary dentition [10-14]. For pediatric patients, CBCT offers distinct advantages over standard CT, one of which is a shorter scan time, which helps to reduce children's anxiety [15-19]. Cone-beam computed tomography (CBCT) yields much less distorted and much larger images. Few in vivo studies have assessed primary tooth obturation using CBCT [12-14]. Therefore, it is of interest to evaluate four distinct obturating methods: endodontic pressure syringe, reamers, Lentulo spirals driven through slow-speed handpiece, and incremental filling technique utilising zinc oxide eugenol (ZOE) paste to serve as obturating material in decidous teeth. Thus, it of interest to determine the effective obturation-methodology using CBCT.

## Methods and Materials:

The baseline test one-way analysis of variance (ANOVA) with a power value of 80% and a coefficient of effect of 0.40, α = 0.05, was used to determine the size of the sample employing G* power 3.1.9.2 software. The number of samples was calculated as 158 and rounded to 160. 160 root canals of deciduous teeth from sixty collaborative, healthy kids of both males and females ageing between 4 years to 9 years were chosen by block random assignment.

## Inclusion:

[1] Kids with a past history of pain

[2] bleeding following coronal pulp removal

[3] inter-radicular radiolucency being present

## Exclusion

[1] teeth that are irreversibly damaged

[2] teeth exhibiting external or internal root resorption

[3] Follicular cysts

[4] Lesions known as dentigerous lesions

[5] Children whose health has declined.

Parents were given a thorough explanation of the methodology of the study, approaches to treatment, and possible outcomes. Prior to the commencement of the therapy, standardized intraoral periapical radiographs (IOPAR) were obtained. A local anaesthetic was given, and the teeth were isolated using a rubber dam. A pulpectomy procedure was carried out. An uncontaminated round bur was inserted through the pulp chamber using a high-speed air-rotor. The coronal portion of the pulp was cut off using a spoon excavator. Radio-visiography (RVG) was performed to verify the results and determine the operational length at one mm less than the apex following radicular pulp removal. Each and every one of the primary tooth root canals was inspected for apical patency using #10 K-file (Densply, India).

The canal was cleaned and shaped using the #15 K-file (Densply, India) initially, and then the Kedo SG Paediatric rotary file system. Irrigation was performed twice after utilising each file: one time with regular saline and another time with 5.25 percent sodium hypochlorite (1 mL). Following that, absorbent paper points were placed 1 mm below the radiography apex to dry the canals. The selected teeth were all treated in the same way. Four categories of forty root canals each were allocated at random applying the block randomization approach. These canals were sealed using four different obturating techniques: endodontic pressure syringe, reamers, Lentulo spirals driven through slow-speed handpiece, and incremental filling technique.

ZOE was selected as the obturating material in the current investigation. By following the procedure outlined by Aylard and Johnson 7, a standardized combination consisting of zinc oxide (ZnO) along with eugenol was made ready for obturation. The mixture was prepared with 1 gm of ZnO and 0.4 mL of eugenol for use in incremental filling technique, reamers and lentulo spirals attached on the rotary handpiece. Approximately, 1 gm of ZnO and 0.275 mL of eugenol was used for obturation with an endodontic pressure syringe.

## Category 1:

## Incremental filling technique:

A thick ZOE paste was introduced to the canal using a canal-sized plugger with a stopper. The endodontic plugger's length was equal to the root canal's planned operational length minus two millimetres. The blocks each measuring 2 mm were added in increments as long as the canal was obturated up to the cervical region.

## Category 2:

## Endodontic pressure syringe:

The bio-mechanically prepared primary tooth root canals in this category were obturated with ZOE paste having creamy consistency in accordance with the manufacturer's instructions by employing an endodontic pressure syringe, (pulpdent, root canal pressure syringe).

## Category 3:

## Lentulo spiral:

With a slow-speed contra-angle handpiece (1000 rpm) carrying a 21 mm Lentulo spiral in size 30, the ZOE paste was inserted into the root canals. The Lentulo spiral was coated with ZOE paste, inserted into the canal, flipped clockwise, and removed while the device was still rotating. Until the biomechanically prepared root canal was completely filled with cement, the procedure was repeated.

## Group 4:

## Reamer technique:

A reamer coated with ZOE was utilised in conjunction with vibration. This procedure was carried out five to seven times, using a rubber stopper to ensure the right working length.

Glass ionomer cement is used in the restoration of teeth. The post-obturation evaluation was conducted using CBCT (Carestream Cs 9300), which is produced by Carestream Health Inc. in France. Following the patient's fitting with a thyroid collar along with lead apron, post-obturation scans were performed in the pedo mode. The images were analysed using software called CS 3D imaging. The quality of the obturation was assessed using the criteria put forth [17].

[1] Under-filling (score 1): the canal is filled more than 2 mm below the apex.

[2] Ideal filling (score 2): canal filling that ends at the radiographic apex or less than 2 mm from it.

[3] Overfillin - filling past the root apex (score 3).

The presence or absence of voids and their quantity in the coronal one third, middle one third, and apical one-third were used to evaluate them. The quality of the obturation was assessed by two independent investigators who were not informed of the group assignment or the obturation method. The score with the lowest possible score was selected when there is a dispute.

## Statistical analysis:

In order to compare the various obturating approaches, the data were statistically analyzed using SPSS 18 software. Two tests were used to analyse the data: the Chi-square test was used to analyse voids, and the Mann-Whitney U test was used to evaluate the filling of the obturated canals.

## Results:

It was observed that handpiece driven lentuspirals helped in optimum obturation in high percentage of root canals. Low percentage of optimally filled root canals was observed in reamers technique. Moreover, percentage of under filled root canals was high in lentuspirals technique of obturation. On the other hand percentage of overfilled root canals was high in endodontic pressure syringe and reamers obturation technique. The variations were statistically significant (Table 1).

## Coronal one third:

Voids were observed in 2.5%, 5.0%, 5.0% and 7.5% of root canals in handpiece driven lentuspirals, endodontic pressure syringe, incremental technique and reamer based obturation technique respectively. It was observed that voids were low in handpiece driven lentuspirals and high in reamer based obturation technique.

## Middle one third:

Voids were observed in 5.0%, 7.5%, 7.5% and 10 % of root canals in handpiece driven lentuspirals, endodontic pressure syringe, incremental filling technique and reamer based obturation technique respectively. It was observed that voids were low in handpiece driven lentuspirals and high in reamer based obturation technique.

## Apical one third:

Voids were observed in 2.5%, 7.5%, 7.5% and 10 % of root canals in handpiece driven lentuspirals, endodontic pressure syringe, incremental technique and reamer based obturation technique respectively. It was observed that voids were low in handpiece driven lentuspirals and high in reamer based obturation technique. It was observed that the percentage of voids in all obturation technique was low (2.5% to 10%) as shown (Table 2).

## Discussion:

Based on radiographic evaluations, studies with varying levels of success have examined the standard of obturation achieved by different obturation techniques [20-21]. The optimal obturation methodology for depth of fill was lentulo spiral [18-19, 22,23,24]. In terms of the quality of obturation, another study state that there is no appreciable distinction between the pressure syringe as well as the Lentulo spiral [15-21]. One of the shortcomings associated with conventional radiography is that it is unable to assess the degree of obturation in all three dimensions [12- 16].

The greatest percentage of optimally filled canals was found with NaviTip along with endodontic pressure syringe, compared with Lentulo spiral and insulin syringe [22-25]. A study to evaluate the depth-of-fill and volume percentage of obturation and voids in primary teeth's root canals (CBCT) is known [8]. The approach that combined the Lentulo spiral and the NaviTip system showed high percentage of canals that were filled to their ideal state and the greatest percentage of volume [8- 10].

The endodontic pressure syringe technique yielded the best-filled canals when contrasted with the other obturation procedures. This could be because the plunger system of the endodontic pressure syringe is much better at providing the much higher pressure required for dispensing the thicker ZOE mixture [4-6]. A pressure syringe for endodontics was created by Greenberg and colleagues. Using a device, a standardized mixture is inserted into the canal under regulated pressure in this method. This set of tools includes a threaded plugger, threaded needle, syringe barrel and wrench [7-10]. Until wall resistance was felt, the needle was inserted into the canal. When the canal is filled at the orifice with zinc oxide eugenol paste, the needle was slowly withdrawn at intervals of 3 mm with each quarter turn of the screw. The root canal can be instrumented using the 13-30 gauge needles, which matches the largest endodontic file [11-14].

A study [9,22] reported the volume of canal overfill, the voids, along with the extent of the fill. The endodontic pressure syringe and tuberculin syringe work better. It was also found that the endodontic pressure syringe performed a better job of controlling the ZOE cement's extrusion [6- 10]. Endodontic pressure syringes produced better results with more ideal obturation method in terms of obturation length [5]. However, there were obvious gaps in the obturation [12-16,22]. The filling effectiveness of several obturating techniques in primary molars is known. The Lentulo spiral produced the best results in terms of canal fill depth, and while the NaviTip syringe was adequately successful at eliminating voids and creating a tight apical seal [18-25]. Another study evaluated the efficacy of different obturation methods utilising a CBCT scan. The five groups whose primary teeth were examined, the Lentulo spiral attached to the hand piece offered the best CBCT obturation technique [22]. Bacterial contamination may arise from micro-leakage in primary teeth with empty spaces in the obturation of root canal [12- 16]. Vacuum spaces along the entire length of the root canal or in the apical region or coronal region may raise the risk of disappointment. In the current analysis, the least number of voids is observed in all procedures [17-19]. This could be the result of properly isolating the teeth with a rubber dam, properly shaping and cleaning the root canals, drying them with paper points, standardizing the ratio of powder to liquid, the material's working properties, the phase of root resorption, and technique proficiency [20- 23].

## Conclusion:

Hand-piece driven lentu-spirals helped in optimum obturation in high percentage of root canals. Low percentage of optimally filled root canals was observed in reamers technique. Moreover, under filled root canals was low in lentu-spirals technique of obturation. However, overfilled root canals were high in endodontic pressure syringe and reamers obturation technique.

## Figures and Tables

**Table 1:** Data regarding proportion filling status of different root canal in different obturation techniques

	**Category 1 (incremental filling technique)**	**Category 2 (endodontic pressure syringe)**	**Category 3**	**Category 4**
			**(lentuspirals driven by handpiece)**	**(reamers)**
Underfilled n (%)	10 (20.50%)	11 (20.25%)	9 (20.25%)	13(35.25%)
Optimum filled n (%)	28(70.00%)	24 (60.00%)	29 (74.75%)	23 (54.75%)
Overfilled n (%)	2 (5%)	5 (10.25%)	2 (5%)	4 (10%)
P value		0.02		

**Table 2:** Data showing comparative incidence of voids in root canals in different obturation techniques

	**Coronal**				**Middle**				**Apical**			
**Groups**	1	2	3	4	1	2	3	4	1	2	3	4
**Voids present n (%)**	2 (5.00)	2 (5.00)	1 (2.5)	3-7.5	3-7.5	3 (7.5)	2(5.0)	4(10)	3(7.5)	3 (7.5)	1(2.5)	4(10)
**Voids absent n (%)**	38-95	38-95	39 (97.5)	37-92.5	37-92.5	37-92.5	38	36-36	37 (92.5)	37-92.5	39 (97.5)	36 (90)
**Chi square**		2.049				1.39				1.039		
**P value**		0.001				0.001				0.001		

## References

[1] Dandashi MB (1993). Pediatr Dent..

[2] Nagar P (2011). J Dent Oral Biosci..

[3] Akhil JEJ (2019). Clin Oral Investig..

[4] Nagaveni NB (2017). J Indian Soc Pedod Prev Dent..

[5] Hiremath MC (2016). J Nat Sci Biol Med..

[6] Chandrasekhar S (2018). J Indian Soc Pedod Prev Dent..

[7] Singh A (2017). Pediatr Dent..

[8] Sijeria P (2018). Int J Clin Pediatr Dent..

[9] Gandhi M (2017). J Clin Diagn Res..

[10] Khubchandani M (2017). Eur J Gen Dent..

[11] Dhillon JK (2013). J Pediatr Dent..

[12] Kumar M (2015). J Int Oral Health..

[13] Aylard SR (1987). Paediatr Dent..

[14] Nezam S (2021). Int J Clin Pediatr Dent..

[15] Mahajan N (2015). Int J Med Dent Sci..

[16] Vashista K (2015). Int J Clin Pediatr Dent..

[17] Coll JA (1996). Pediatr Dent..

[18] Singh R (2015). J Clin Pediatr Dent..

[19] Nagarathna C (2018). Contemp Clin Dent..

[20] Coll JA (1988). Pediatr Dent..

[21] Nagaveni NB (2017). J Clin Pediatr Dent..

[22] Ali SM (2023). Int J Clin Pediatr Dent..

[23] Allen KR (1979). Aust Dent J..

[24] Memarpour M (2013). Pediatr Dent..

[25] Bahrololoomi Z (2015). J Dent Mater Tech..

